# Neuroimaging evidence supporting a dual-network architecture for the control of visuospatial attention in the human brain: a mini review

**DOI:** 10.3389/fnhum.2023.1250096

**Published:** 2023-09-28

**Authors:** Annalisa Tosoni, Paolo Capotosto, Antonello Baldassarre, Sara Spadone, Carlo Sestieri

**Affiliations:** Department of Neuroscience, Imaging and Clinical Sciences (DNISC) and ITAB, Institute for Advanced Biomedical Technologies, G. d’Annunzio University of Chieti-Pescara, Chieti, Italy

**Keywords:** visuospatial attention, dorsal attention network, ventral attention network, orienting, reorienting, neglect

## Abstract

Neuroimaging studies conducted in the last three decades have distinguished two frontoparietal networks responsible for the control of visuospatial attention. The present review summarizes recent findings on the neurophysiological mechanisms implemented in both networks and describes the evolution from a model centered on the distinction between top-down and bottom-up attention to a model that emphasizes the dynamic interplay between the two networks based on attentional demands. The role of the dorsal attention network (DAN) in attentional orienting, by boosting behavioral performance, has been investigated with multiple experimental approaches. This research effort allowed us to trace a distinction between DAN regions involved in shifting vs. maintenance of attention, gather evidence for the modulatory influence exerted by the DAN over sensory cortices, and identify the electrophysiological correlates of the orienting function. Simultaneously, other studies have contributed to reframing our understanding of the functions of the ventral attention network (VAN) and its relevance for behavior. The VAN is not simply involved in bottom-up attentional capture but interacts with the DAN during reorienting to behaviorally relevant targets, exhibiting a general resetting function. Further studies have confirmed the selective rightward asymmetry of the VAN, proposed a functional dissociation along the anteroposterior axis, and suggested hypotheses about its emergence during the evolution of the primate brain. Finally, novel models of network interactions explain the expression of complex attentional functions and the emergence and restorations of symptoms characterizing unilateral spatial neglect. These latter studies emphasize the importance of considering patterns of network interactions for understanding the consequences of brain lesions.

## 1. Introduction

**Figure 1:**
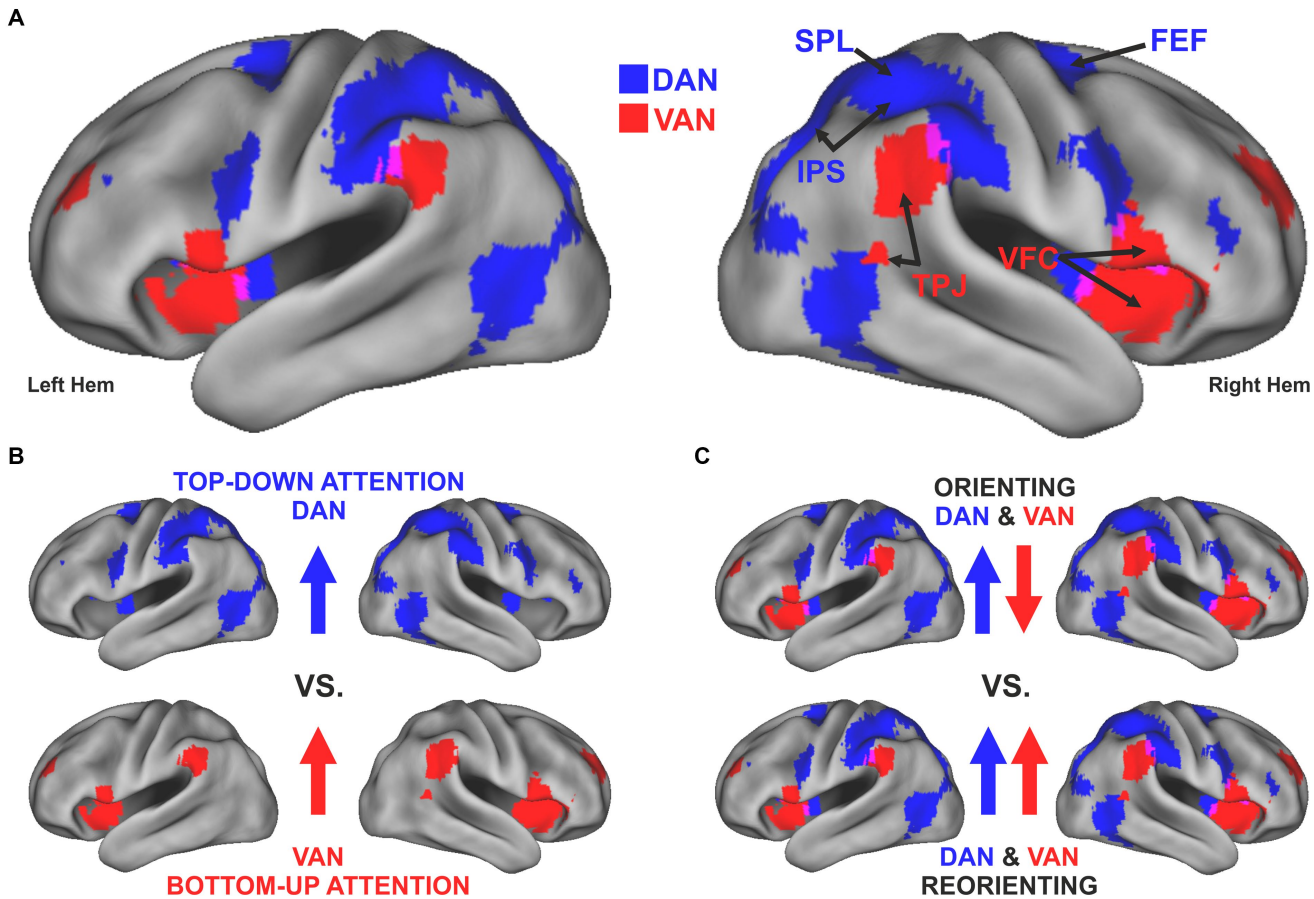
Evolution of a dual-network model for the control of visuospatial attention. **(A)** The topography of the dorsal (blue) and ventral (red) attention network, defined by resting state functional connectivity derived from a set of task-based key regions of these networks (Hacker et al., 2013). The maps are superimposed on an inflated map of both hemispheres obtained using Caret Software v. 5.65 (Van Essen, 2005). The main nodes of each network are highlighted in the left hemisphere. **(B)** The original model of a dual network architecture (Corbetta and Shulman, 2002) emphasized the distinction between *top-down* (e.g., endogenous) attentional control, implemented by the DAN, and *bottom-up* or stimulus-driven (e.g., exogenous) control, implemented by the VAN. **(C)** Subsequent studies (reviewed in Corbetta et al., 2008) have shown that the anatomical DAN/VAN distinction does not easily map onto a simple functional top-down/bottom-up distinction. The DAN and the VAN exhibit opposite activity when attention is voluntarily *oriented* to a stimulus’ location or feature based on either endogenous or exogenous cues. Instead, the VAN is transiently recruited, along with the DAN, during the *reorienting* of attention and the detection of a target, especially if salient or unexpected.

Visuospatial attention can be guided in a *top-down* fashion by internal goals and expectations or in a *bottom-up* manner by the detection of salient and behaviorally relevant stimuli. Neuroimaging research in the last quarter century has gathered evidence for a dual-network architecture involved in these two types of attentional control (Corbetta and Shulman, 2002). Accordingly, the focus of attention is determined by the dynamic interaction between a *dorsal* attention network (DAN), which includes bilateral regions of the superior parietal lobule (SPL), the intraparietal sulcus (IPS), and the frontal eye field (FEF), and a *ventral* attention network (VAN), which includes regions of the temporoparietal junction (TPJ) and the ventral frontal cortex (VFC) (Figure 1A). The two networks were originally identified based on task-evoked activity using positron emission tomography (PET) and functional magnetic resonance imaging (fMRI) and their anatomical segregation has been initially associated with a fundamental distinction between top-down and bottom-up attention (Corbetta and Shulman, 2002) (Figure 1B). The same networks have been later identified by resting-state functional connectivity studies using fMRI (Fox et al., 2006) or magnetoencephalography (MEG) (de Pasquale et al., 2012).

The scope of the present mini-review is to summarize recent findings on the neurophysiological mechanisms implemented in both networks in normal and pathological conditions. It is now clear that the DAN/VAN distinction does not easily map onto a simple top-down/bottom-up, as initially hypothesized. The DAN exhibits sustained activity when attention is voluntarily oriented to a stimulus’ location or feature based on either endogenous or exogenous cues, consistent with a general role in the control of visuospatial attention. Instead, the VAN is deactivated during the sustained orienting of attention but transiently recruited, along with the DAN, during the reorienting of attention and the detection of a target, especially if salient or unexpected (Figure 1C). Several studies have gradually elucidated the anatomical and functional properties of the two networks and identified frequency-specific correlates of attentional orienting and reorienting. Finally, novel models of network interactions explain the expression of complex attentional functions as well as the emergence and restorations of symptoms characterizing unilateral spatial neglect. We conclude this mini-review by highlighting several outstanding issues.

## 2. The dorsal attention network

Data obtained with a variety of experimental paradigms support the crucial role of the DAN in the representation of an *attentional set* and the appropriate selection of task-relevant stimuli and responses (Corbetta and Shulman, 2002). The DAN is involved in orienting attention to locations, features, or objects (Wojciulik and Kanwisher, 1999; Slagter et al., 2007), with or without eye movements (Corbetta, 1998; Corbetta et al., 1998). Its activation pattern is sustained over long intervals (Corbetta et al., 2002; Sylvester et al., 2007) and is predictive of behavioral performance (Pessoa and Padmala, 2005; Sapir et al., 2005). Stimulus location and behavioral relevance in the DAN are coded within several retinotopic maps (Wandell et al., 2007; Silver and Kastner, 2009), updated by either endogenous or exogenous information (Kincade et al., 2005; Serences et al., 2005). Furthermore, the DAN encodes spatial locations in multiple reference frames and motor effectors (Cohen and Andersen, 2002; Ptak, 2012), allowing the formation of sensorimotor associations in perceptual decision-making (Tosoni et al., 2008, 2014, 2017).

**Figure 2:**
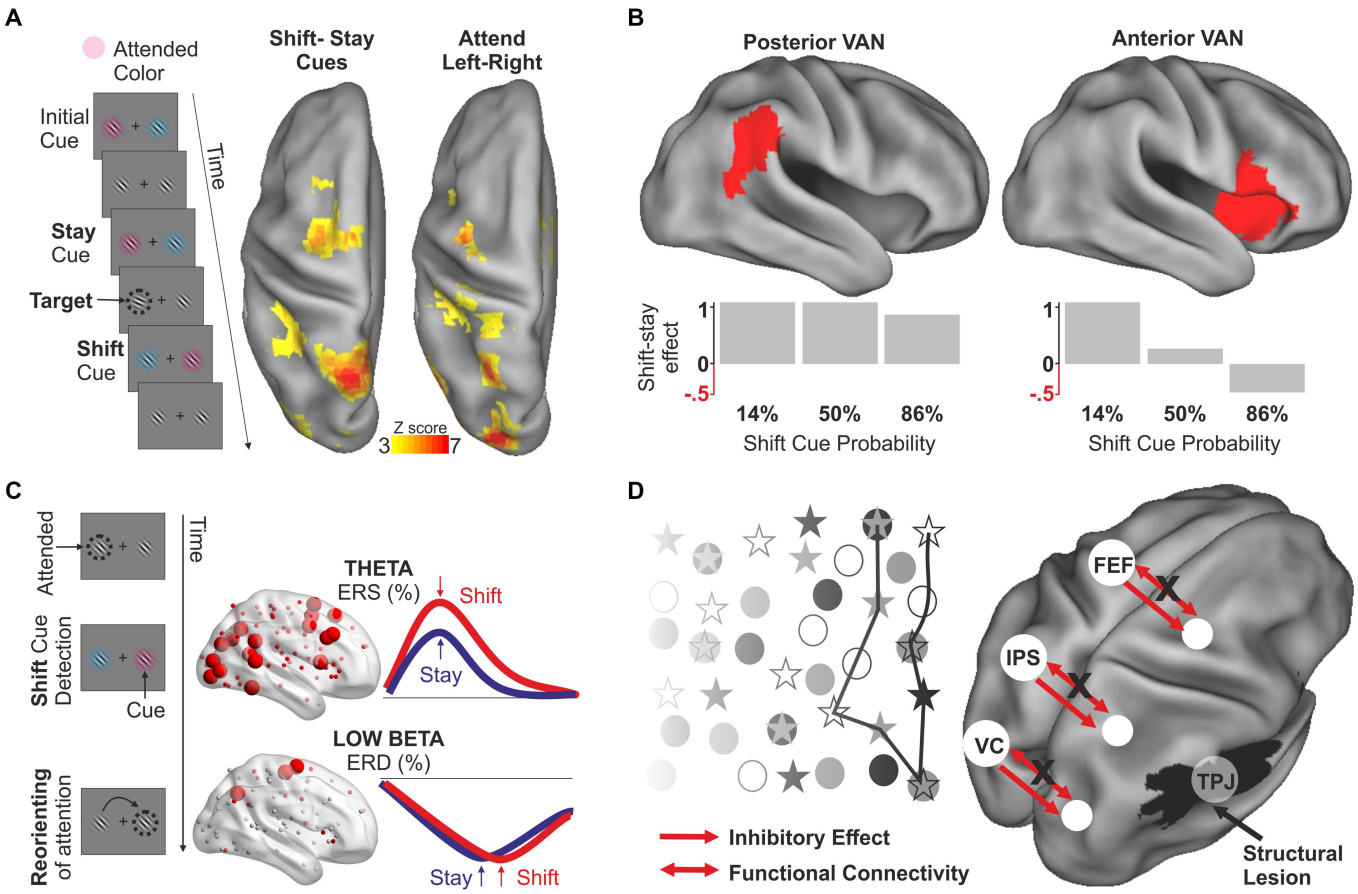
Functional distinctions within the DAN and the VAN and network interactions in normal and abnormal conditions. **(A)** In the task employed by Spadone et al. (2015), attention is continuously allocated toward a peripheral spatial location to detect randomly appearing targets. Stay cues indicate to maintain attention at the same location whereas shift cues indicate the need to reorient attention to the opposite hemifield. Attentional reorienting, assessed through the difference between shift and stay cues, involves medial DAN regions, whereas more lateral DAN regions respond when holding attention at contralateral locations. **(B)** Using a similar continuous task, Shulman et al. (2009) showed that the reorienting response in the posterior and anterior nodes of the VAN differs in relation to the frequency of the reorienting. Whereas the reorienting effect in the posterior nodes is observed regardless of whether shift cues are rare or frequent, the anterior nodes exhibit a reorienting effect only when shift cues are unexpected. The figure is only indicative of the effect, for exact information about the ROIs and the shift-stay effect as a function of cue probability refer to Table 1 and Figure 5 of the original article. **(C)** Using the same paradigm depicted in panel **(A)**, Spadone et al. (2021a) investigated the frequency-specific modulations unfolding in time during the reorienting of attention. This study showed that the detection of the shift cue indicating the need to reorient attention is associated with a widespread increase of power in the theta band which is observed in both the DAN and the VAN, possibly reflecting an early alert/reset signal. This effect is followed by a sustained desynchronization in the low-beta band which is selectively observed in the DAN and correlated with behavioral performance. This second effect is likely associated with the implementation of control signals operating the actual reorienting. **(D)** Example of network interaction in the pathophysiology of spatial neglect (original model in Corbetta and Shulman, 2011). According to the hypothesis, structural damages to the VAN not only cause direct non-spatial deficits associated with the functions of the VAN but also indirect dysfunction of anatomically intact regions of the DAN that control the orienting of visuospatial attention. In particular, lesions to the VAN cause an imbalance between the activity of the two hemispheres favoring the left hemisphere but also a decrease of connectivity between DAN regions of the affected hemisphere, leading to a contralateral attentional bias (indicated by behavior in cancelation test).

The role of the DAN in attentional orienting has been traditionally studied using variants of the Posner cueing paradigm (Posner et al., 1980), in which attentional reorienting coincides with the detection of a target presented at unexpected locations. However, reorienting can be distinguished from target detection and/or motor execution by presenting cues indicating to either maintain or shift peripheral attention in anticipation of a target (Yantis et al., 2002; Shulman et al., 2009). Using this paradigm, several studies have identified a region in the medial superior parietal lobule (mSPL) that exhibits a robust, transient activation for shifting between locations (Yantis et al., 2002; Kelley et al., 2008), objects (Serences et al., 2004), modalities (Shomstein and Yantis, 2004), and categorization rules (Chiu and Yantis, 2009). A functional distinction has also been described between transient shift-related signals in the mSPL and sustained, spatially-selective signals in more lateral IPS and FEF regions associated with holding attention at the contralateral location (Shulman et al., 2009; Tosoni et al., 2012; Spadone et al., 2015) (Figure 2A). This functional specialization has received further support from studies employing an integrated transcranial magnetic stimulation (TMS)-fMRI-EEG approach. In particular, lateral and medial DAN regions are associated with different interference (i.e., behavioral) effects from TMS (Capotosto et al., 2013) but also with oscillatory activity in different low frequencies (alpha and delta rhythms) (Capotosto et al., 2015). A recent MEG study demonstrated that the above functional-anatomical segregation is associated with an increase in information flow in the beta band involving more medial (for shifting) and lateral (for holding) parietal nodes of the DAN (Spadone et al., 2021b).

According to neurophysiological models of attention (Desimone and Duncan, 1995; Kastner and Ungerleider, 2000), the DAN controls the orienting of attention by modulating the activity of sensory cortices, a hypothesis initially supported by microstimulation studies in monkeys (Moore and Fallah, 2001; Moore and Armstrong, 2003) and more recently corroborated by human studies. For example, by combining TMS with fMRI, Ruff and colleagues have shown that TMS over DAN regions modulates fMRI activity in the visual cortex as well as psychophysical sensitivity (Ruff et al., 2006, 2009). Using TMS in combination with electroencephalographic (EEG) recordings, Capotosto and colleagues demonstrated that the stimulation of DAN regions affects the preparatory alpha activity during target anticipation, impairing behavioral performance (Capotosto et al., 2009, 2011). Evidence for modulatory effects, compatible with a mechanism of top-down influence from DAN to visual cortex and predictive of behavioral performance, has been also obtained through analysis of causality on fMRI time series (Bressler et al., 2008; Vossel et al., 2012). Notably, these modulatory influences occur without a dramatic reorganization of the network architecture (Spadone et al., 2015) and reflect a purely endogenous, sustained process (Meehan et al., 2017).

In the last 15 years, the neural mechanisms of visuospatial attention have also been successfully studied at high temporal resolution using EEG and MEG. A first notable finding has been the discovery that the topography of task-evoked modulations observed with fMRI is recapitulated by the topography of slow (<0.1 Hz) coherent fluctuations of band-limited power (BLP) recorded with MEG across multiple frequencies (Betti et al., 2013; Favaretto et al., 2021). Focusing on the orienting of attention, two main oscillatory mechanisms in the parieto-occipital cortex have been identified: a pre-stimulus event-related desynchronization (ERD) in alpha and beta bands, indexing preparatory attention, and a post-stimulus event-related synchronization (ERS) in the gamma band, a putative correlate of top-down modulation of sensory information boosting behavioral performance (Jensen and Mazaheri, 2010; Foxe and Snyder, 2011; Gregoriou et al., 2015). Moreover, the DAN appears to modulate visual regions through frequency-specific inter-regional synchronization (Siegel et al., 2008), in line with the current hypothesis on the communication between neuronal populations (Fries, 2005, 2015). A study using electrocorticography (ECoG) has further revealed that the DAN becomes selectively phase-modulated at even lower frequencies (delta, theta) during attentional orienting (Daitch et al., 2013), which might reflect the role of these rhythms in attentional sampling (Fries, 2015).

## 3. The ventral attention network

As outlined in the introductory section, the VAN shows two main functional properties. Firstly, the network is *deactivated* during the sustained orienting of voluntary attention to a stimulus’ location or feature (Shulman et al., 2003), acting as a filter that prevents inappropriate responses to irrelevant stimuli (Shulman et al., 2007). Secondly, the VAN is recruited by the detection of behaviorally-relevant stimuli, particularly when unexpected (Marois et al., 2000; Stevens et al., 2005) or presented in unattended locations (Arrington et al., 2000; Corbetta et al., 2000). On these bases, the VAN has been initially conceptualized as a “circuit-breaker” system for interrupting activity in the DAN when unexpected or novel stimuli are detected (Corbetta and Shulman, 2002). However, later studies have demonstrated that the VAN is particularly tuned to behavioral relevance rather than mere sensory salience (de Fockert et al., 2004; Serences et al., 2005). Also, its relatively late onset of activation (Mangun and Hillyard, 1991; Luck et al., 1994) compared to regions of the DAN (Evdokimidis et al., 2001; Sestieri et al., 2008) and its weak spatial selectivity (Jack et al., 2007) appears inconsistent with a role in the initiation of a reorienting response (Corbetta et al., 2008). Finally, the VAN encodes transient signals during task transitions (Fox et al., 2005) and is involved in various aspects of social cognition, such as during theory of mind (ToM) tasks (Mitchell, 2008; Geng and Vossel, 2013), features that suggest a more general role in resetting ongoing activity and switching between internally- and externally- directed attention (Corbetta et al., 2008).

Another distinctive property of the VAN concerns its hemispheric lateralization. Although spatial attention has been traditionally considered a globally right-lateralized function, a clear right hemispheric dominance has only been demonstrated for the VAN (Arrington et al., 2000; Corbetta et al., 2000). Notably, an fMRI study on this issue has found evidence that the right hemispheric dominance in the VAN is observed for stimulus-driven shifts of attention and target detection (Shulman et al., 2010), while a more bilateral activation is observed when contrasting activity during invalid vs. neutral, compared to valid, trials of a Posner task (Doricchi et al., 2010). Supporting evidence for the right lateralization of the VAN comes from functional connectivity studies (Fox et al., 2006; Liu et al., 2009), especially concerning the TPJ region (Kucyi et al., 2012), whereas a more bilateral pattern of functional connectivity is typically observed in the DAN (Fox et al., 2006).

Some studies have also demonstrated functional segregation in the VAN by distinguishing between signals related to reorienting attention and the violation of expectation. Whereas these processes are intrinsically intertwined in the oddball (Marois et al., 2000; Stevens et al., 2005) and Posner cueing task (Arrington et al., 2000; Corbetta et al., 2000), a factorial manipulation of these two factors has been conducted within an RSVP paradigm (Shulman et al., 2009). Whereas frontal nodes were selectively activated when attentional reorienting was unexpected, possibly reflecting a specific role in response inhibition (Aron et al., 2004), the TPJ exhibited independent modulations by attentional reorienting and violation of expectation, suggesting an additive role of this region in task switching/resetting and control of expectations (Figure 2B). Both mechanisms are compatible with the hypothesis that TPJ activity relates to single unit activity in the locus ceruleus/norepinephrine system and with network reorganization triggered by behaviorally relevant stimuli (Aston-Jones and Cohen, 2005; Bouret and Sara, 2005).

Several studies conducted in the last decade have also started to question the evolution of the VAN across species. In contrast to key regions of the DAN, for which homolog areas have been well described in primates (e.g., Colby et al., 1996; Thompson et al., 1997), the presence of a VAN in the monkey brain is still debated. For example, although functional equivalents of both resting state networks have been described (Mantini et al., 2012, 2013), the network homology was lower than expected (see also Mars et al., 2012). At the anatomical level, large interspecies differences have been observed in the organization of the ventral branch of the superior longitudinal fasciculus (SLF) that connects regions of the VAN (Hecht et al., 2015). Finally, a study directly comparing fMRI activity in humans and monkeys performing the same attention task found differences in the structure and organization of regions of the DAN in the two species (Patel et al., 2010) and, more importantly, no functional homolog of the TPJ in macaques (Patel et al., 2015). Considering the large cortical surface expansion of this area across primate evolution, a novel attention network might have emerged to satisfy human-specific evolutionary pressures (Sliwa and Freiwald, 2017). Specifically, the TPJ-pSTS could represent a key hub of a human-specific visual processing stream that merges information obtained from the exploration of the external sensory world with internally generated models of social factors (Patel et al., 2019).

## 4. Network interaction in normal and abnormal cognition

As outlined in the introduction, a dynamic interaction is assumed between DAN and VAN to achieve flexible control of visual attention (Vossel et al., 2014). However, the mechanisms supporting this interaction are still largely unknown. Evidence for a DAN-VAN interaction comes from analyses of causal relationships between networks (Wen et al., 2012; Leitao et al., 2015) and from studies of functional connectivity that identified a region of the middle frontal gyrus (MFG) that correlates with both DAN and VAN (Fox et al., 2006; He et al., 2007). These data suggest that the MFG might represent a site of convergence allowing network interaction. Supporting evidence for the role of lateral prefrontal regions in the functional integration between the VAN and the DAN comes from an fMRI study on the surprise-induced blindness effect (Asplund et al., 2010). Another example of a complex behavior requiring network interaction is the reorienting response based on incoming sensory information. A recent MEG study using a modification of the continuous shift/stay paradigm has characterized the complex pattern of frequency-specific modulations that unfold over time during the reorienting of attention (Spadone et al., 2021a). In this study, a widespread increase of power in the theta band [see also (Proskovec et al., 2018) for analogous results in a Posner-like paradigm] was first observed in both the DAN and the VAN, possibly reflecting an early alert/reset signal triggered by the detection of the shifting cue. Then, the DAN is thought to implement the actual reorienting through a sustained desynchronization in the low-beta band (Figure 2C).

Other studies have focused on mechanisms of network interaction during unilateral spatial neglect, a neurological syndrome caused by lesions to the right hemisphere and characterized by a failure to attend and respond to stimuli presented in the contralesional field (Vallar, 1998; Mesulam, 1999). The emergence of a major deficit in attentional orienting is difficult to explain based on a simple anatomo-clinical correlation (Deuel and Collins, 1983) since the syndrome is typically associated with lesions occurring at/near the VAN (Mort et al., 2003; Karnath et al., 2004). FMRI evidence indicates instead that the syndrome emerges from the interaction between the two attentional networks (Corbetta et al., 2005; He et al., 2007). According to a recent model (Corbetta and Shulman, 2011), structural damages to the VAN have two main consequences. Firstly, they directly cause non-spatial deficits (e.g., general slowness) that reflect the involvement of the VAN in arousal, reorienting, and detection of behaviorally relevant stimuli. Second, through associated damages of white matter fibers connecting the two networks, they also cause dysfunction of anatomically intact regions of the DAN that control the orienting of visuospatial attention. In particular, it has been demonstrated that damages to the VAN cause a hypoactivation of the right hemisphere, a reduction of the VAN-DAN cross-network interactions, and a decrease of connectivity between DAN regions of the affected hemisphere (Corbetta et al., 2005; He et al., 2007) (Figure 2D). In turn, this would produce an imbalance between the activity of the two hemispheres favoring the left hemisphere, both at rest (He et al., 2007) and during a task (Corbetta et al., 2005), leading to a contralateral attentional bias. Consistent with this model, the resurgence of the inter-hemispheric balance of activity within the DAN explains the partial recovery from the more obvious spatial deficits (Corbetta et al., 2005; He et al., 2007; Rengachary et al., 2011).

These latter studies emphasize the importance of considering the abnormal patterns of anatomical and functional connectivity, in addition to focal damage, for understanding the behavioral consequences of brain lesions (Baldassarre et al., 2016b; Siegel et al., 2017, 2022). For example, recent experimental work highlights the importance of *physiological abnormalities* in large-scale functional interaction between *anatomically intact* cortical regions for the development of neglect symptoms. Specifically, the severity of neglect behavioral symptoms is associated with the reduction of the interhemispheric connectivity within the DAN and with the increase of intrahemispheric connectivity between the DAN and normally anticorrelated networks (Baldassarre et al., 2014, 2016a). Crucially, a longitudinal study further indicates that recovery from neglect symptoms is associated with the restoration of the normal pattern of functional connectivity (Ramsey et al., 2016), suggesting possible directions for neurological interventions. Indeed, recent neuroimaging reports described dysfunctions of task-evoked and intrinsic activity within the DAN in posterior cortical atrophy (Veldsman et al., 2019), traumatic brain injury (Mallas et al., 2021) as well as mild cognitive impairment and Alzheimer’s disease (Zhang et al., 2015), suggesting a key role of this network in the pathophysiology of different brain disorders.

## 5. Discussion

Attention is a core property of all perceptual and cognitive operations, modulating both externally- and internally-generated information (Chun et al., 2011). The functional properties of the DAN and the VAN and their functional interactions are thought to reflect a general mode of brain organization regardless of the particular sensory modality under consideration (even if major information comes from the field of vision), suggesting the existence of supramodal systems (Macaluso, 2010). However, the role of the two networks in other domains, such as orienting attention to long-term memories, is still debated (Cabeza et al., 2008; Sestieri et al., 2017). Another interesting, but rather unexplored field of investigation, concerns the plasticity of the connectivity between the attention networks and sensory cortices. Perceptual learning modulates the connectivity between the DAN and visual cortex in a spatially-selective manner (Lewis et al., 2009; Liu et al., 2010). Future work should test whether this is a general mechanism resulting from intense training and expertise. Another crucial issue concerns the position of the two networks within the brain’s functional architecture and the degree to which the antagonistic relationship between the DAN and the default mode network (DMN) is coordinated by a higher-order neural system (Sestieri et al., 2014), such as the cingulo-opercular network (Dosenbach et al., 2006; Power and Petersen, 2013). This hypothesis should be tested using analyses of causal relationships (e.g., Sridharan et al., 2008; Higo et al., 2011), combined BOLD-fMRI-ECoG recording achieving high spatial and temporal resolution (e.g., Daitch et al., 2013), and analyses of the pattern of connectivity at rest and during task execution (e.g., Cole et al., 2014; de Pasquale et al., 2017).

## Author contributions

AT drafted the manuscript and organized the contributions from different co-authors. PC edited the manuscript sections on TMS and neurophysiological findings on DAN-VAN attention modulations. AB edited the manuscript sections on attentional networks dynamics in the neglect syndrome and other brain disorders. SS edited the manuscript sections on MEG findings on DAN-VAN dynamic interactions. CS managed the general writing and organization of the manuscript. All authors contributed to the article and approved the submitted version.

## Funding

The study was conducted under the framework of the “Departments of Excellence 2023–2027” initiative of the Italian Ministry of Education, University and Research to the Department of Neuroscience, Imaging and Clinical Sciences (DNISC) of the University of Chieti-Pescara.

## Conflict of interest

The authors declare that the research was conducted in the absence of any commercial or financial relationships that could be construed as a potential conflict of interest.

## Publisher’s note

All claims expressed in this article are solely those of the authors and do not necessarily represent those of their affiliated organizations, or those of the publisher, the editors and the reviewers. Any product that may be evaluated in this article, or claim that may be made by its manufacturer, is not guaranteed or endorsed by the publisher.
